# Technical Challenges in the Derivation of Human Pluripotent Cells

**DOI:** 10.4061/2011/907961

**Published:** 2011-06-19

**Authors:** Parinya Noisa, Rangsun Parnpai

**Affiliations:** Embryo Technology and Stem Cell Research Center, School of Biotechnology, Institute of Agricultural Technology, Suranaree University of Technology, 111 University Avenue, Nakhon Ratchasima 30000, Thailand

## Abstract

It has long been discovered that human pluripotent cells could be isolated from the blastocyst state of embryos and called human embryonic stem cells (ESCs). These cells can be adapted and propagated indefinitely in culture in an undifferentiated manner as well as differentiated into cell representing the three major germ layers: endoderm, mesoderm, and ectoderm. However, the derivation of human pluripotent cells from donated embryos is limited and restricted by ethical concerns. Therefore, various approaches have been explored and proved their success. Human pluripotent cells can also be derived experimentally by the nuclear reprogramming of somatic cells. These techniques include somatic cell nuclear transfer (SCNT), cell fusion and overexpression of pluripotent genes. In this paper, we discuss the technical challenges of these approaches for nuclear reprogramming, involving their advantages and limitations. We will also highlight the possible applications of these techniques in the study of stem cell biology.

## 1. Introduction

Pluripotent cells can give rise to any fetal or adult cell types, over 200 specific cell types. Those in contrast to progenitor cells that are able to differentiate into a limited number of cell fates are described as multipotent cells, such as hematopoietic progenitor cells. The first pluripotent human embryonic stem cells (hESCs) were derived from the isolation and culturing of the human inner cell mass (ICM) [1]. The methodology for deriving hESCs has remained the same as the original protocol for the derivation of pluripotent mouse ESCs [2]. According to the first described protocols detailing the propagation of hESCs, the blastocyst's outer trophecodermal layer is first removed by immunosurgery and the ICM is subsequently plated onto gamma-irradiated or mitomycin C-treated mouse embryonic fibroblasts (MEFs) in the presence of high serum concentration. After several days in culture, hESC colonies begin to form [1, 3]. Undifferentiated colonies of most hESCs show a compact morphology with a high nucleus-cytoplasm ratio and retain pluripotent ability in both *in vitro* and *in vivo* experiments. They are able to form embryoid bodies which lead to spontaneous differentiation into three embryonic germ layers [4]. hESCs can also form teratomas when implanted into SCID mice [5, 6], which reflects their *in vivo* differentiation capability. Such teratoma contribute cellular regions representative of all three embryonic germ layers, including gut and glandular epithelium (indicative of endoderm), cartilage, bone and smooth muscle (indicative of mesoderm), and neural epithelium and embryonic ganglia (indicative of ectoderm) [1, 3]. However, due to ethical restrains, cannot be tested in human system. The major limitations of hESC establishment are the availability of donated IVF embryos and ethical restriction in some countries. This has brought about the development of alternative nuclear reprogramming approaches to obtaining human pluripotent cells that are closely resemble to hESCs. It was long thought that when cell differentiates, it loses their plasticity and permanently inactivated gene that is no longer need. Recent findings demonstrated three approaches for nuclear reprogramming which are, (1) somatic cell nuclear transfer, (2) cell fusion, and (3) direct reprogramming of somatic cells by overexpression of hESC transcription factors. However, to improve the success rate of derivation of human pluripotent cells, it is essential to understand the key regulatory network of pluripotency because this knowledge will improve the derivation proficiency and culture conditions of human pluripotent cells. Therefore, this paper intend to describe the basic pluripotency network of human pluripotent cells which is followed by the discussion of technical challenges of the aforementioned reprogramming approaches.

## 2. Pluripotency: The Regulation Mechanisms of Human Embryonic Stem Cells (hESCs)

Self-renewal of hESCs is regulated by both intrinsic and extrinsic factors. Intrinsic factors are transcription factors that are essential for maintaining hESC identity. The best studied intrinsic factors are OCT4, NANOG and SOX2, which play essential roles in both mouse and human ESCs. OCT4, encoded by the POU5F1 locus, is a homeodomain transcription factor of the POU family. OCT4 is necessary for pluripotency, as defined by gene knockout and transgenic experiments in mice [7]. Knockingdown OCT4 by RNAi in hESCs forced them to differentiate into extraembryonic endoderm lineages [8]. Studies have defined several target genes of OCT4. Genes dependent on OCT4 activity for their expression include FGF4 [9], REX1 [10], and Lefty-1 [11] while human chorionic gonadotropin (HCG) is repressed by OCT4 activity [12]. Nanog and SOX2 are also highly expressed in hESCs and dramatically downregulated upon cell differentiation [3, 13, 14]. Like OCT4, NANOG expression appears to be crucial for the maintenance of ICM and hESCs; removal of NANOG results in ICM cells adopting a visceral and parietal endoderm fate while overexpression retards differentiation of hESCs and forces the maintenance of undifferentiated phenotype [15]. Similar to OCT4, SOX2 is important to maintain pluripotent state of hESCs. The deficiency of SOX2 mediated by RNAi is able to cause hESC differentiation toward the trophectoderm [13]. The significant roles of SOX2 in pluripotency have been confirmed by the ability to reprogram human fibroblasts to become pluripotent cells by expression of SOX2 along with OCT4, KLF4, and c-Myc [16]. 

Extrinsic factors, such as growth factor signaling pathways, are also very important for regulating self-renewal of hESCs. However, unlike intrinsic factors, the signaling pathways required for maintaining self-renewal of mouse and human ESCs seem very different. Although both ESCs were originally isolated and maintained by coculture on mitotically inactivated MEF feeder cells, they may require different signals from the feeder cells for retaining their undifferentiation status. Whereas the derivation of mESCs and their propagation in an undifferentiated state requires LIF [17], hESC self-renewal requires FGF2 [3]. One possible reason for this difference may result from the different growth factor receptor expression profiles in mouse and human ESCs. mESCs express leukemic inhibitory factor (LIF) receptor/gp130 receptor complexes, which bind LIF and mediate pluripotency through downstream activation of STAT3 [18]. In contrast, hESCs do not express LIF receptors or gp130 receptors [19]. FGF signaling is thought currently to be the predominant mechanism by which hESC pluripotency is maintained in culture. It was found that undifferentiated hESCs express FGFR1, the cognate receptor for FGF2, more abundantly than differentiated cells [19]. Other FGFRs, including FGFR2, FGFR3, and FGFR4, also appear to be enriched in the undifferentiated hESCs [20]. In addition to FGF signaling, activin/nodal pathway also maintains pluripotency of hESCs through mechanisms in which FGF2 acts as a competence factor [21]. It is hoped that, as the different mechanisms underlying pluripotency of hESCs are unrevealed, essential growth factors, cytokines, and signaling molecules will be discovered, and this could improve the maintenance and derivation efficiency of human pluripotent cells in the simplified, but optimal, systems for prolonged periods of time.

## 3. The Birth of Human Pluripotent Embryonic Stem Cells (hESCs)

In 1998, Thomson et al. reported the first establishment of hESCs from blastocysts of donated IVF embryos [1]. Growth of hESCs on inactivated MEFs or in the presence of MEF-conditioned media introduces the potential of transferring xenobiotic pathogens from the mouse to human tissues. Thus, later, the derivation of hESC lines using human feeder and human serum in order to avoid potential contamination with xenoproteins and xenogenic tissues was reported [22]. These experiments show that new hESC lines can be derived in the xeno-free system, thus permitting their therapeutic application in the future. However, it was an issue of using feeder cells such as the variation of feeder cells, the inconvenience in large-scale production, and the suspicion of molecules produced by feeder cells. Significant progress has been made very recently in understanding how to maintain hESCs in feeder-free environments by virtue of supplementation by various cytokines, either FGF2 alone [23] or in combination with either noggin [24] or activin A [25, 26]. To further apply hESC for clinical lines, it is obviously a priority to eventually refine hESC culture system in order to reach GMP standard, which is including culture conditions, physical environment, facility construction, equipment, and maintenance [27]. One of the key issues causing hESC technology to be useful for cell and tissue therapy in human is the histocompatibility [28]. Recent data support the concept that hESCs and their differentiated progeny possess immune-privileged properties, suggesting that cells derived from hESCs may provide a potential tool for induction of immunotolerance [29]. However, there are only few available hESC lines, which are derived as clinical grade and there is a concern over the genetic stability of hESCs after long-term amplification *in vitro* [30]. More importantly, the ethical debate over the destruction of human embryos has largely prohibited the derivation of large number of hESC lines. The discovery of hESCs opened up the possibility for the application of human pluripotent cells in transplantation therapy, drug screening, and toxicology studies. However, the previously mentioned obstacles must be overcome before such potential can be realized. In another scenario for which the term “personalized pluripotent cells” has been coined, people could use their own somatic cells to be reprogrammed back to the pluripotent cell state. The following message emphasizes on the challenges of the creation of human pluripotent cells by various approaches.

## 4. Somatic Cell Nuclear Transfer (SCNT): The Classical Approach of Nuclear Reprogramming

When a nucleus from differentiated somatic cells, such as skin cells, is transplanted into an enucleated oocyte, nuclear reprogramming is initiated, leading to the generation of an entire individual, which is genetically identical clone of the original somatic cells. Generation of pluripotent cells by SCNT has been well documented in mouse model [31, 32]. It is noted that the process of SCNT is comprised of multiple stages and technical demanding. Principle technique of nuclear transfer involves a somatic donor cell and unfertilized, enucleated oocytes. The nucleus from the somatic donor cells is transplanted into the enucleated oocytes by micromanipulator, leading to union of both components. This reconstructed cell is stimulated to progress embryonic development. Stimulation could be performed by either electrical pulse or chemical agents [33]. The unifying aspect of the reprogramming of somatic nuclei following their transfer into the egg is that the biochemical changes establishing constrains on genetic potential are reversed. The efficiency of this reversal most probably determines the subsequent developmental success of the nuclear transfer embryos. It was realized that nuclear reprogramming by nuclear transfer to enucleated oocytes is an inefficient process. While there are sporadic reports of high efficiency, the overall rate of development to offspring is of the order of 1–3% [34]. The oocytes to use in SCNT could be either* in vivo *or *in vitro* matured cells, but the *in vivo* matured oocytes seem to give a better rate of blastocyst development [35]. The explanation for this event is that oocytes from sexually mature animals are more developmentally competent because they have a better supply of factors that will remodel the nucleus when it is transferred to the nucleus. In addition to the source of oocytes, the origin of donor cells also affects the quality of SCNT-derived embryos. The relatively less differentiated donor cells result in the better outcome of blastocyst development when compared to more differentiated cells [34]. In addition, the longer culturing of donor cells *in vitro* generally causes the decreased development to blastocysts [36]. The possibility for this reason is that the nuclei from less differentiated cells are more plastic and more readily able to remove and replace proteins that affect transcription than nuclei of more differentiated cells. This also correlates with the progressive stabilization of various repressive chromatin structures that assembles as development proceeds [37]. In addition to all those considerations, SCNT-derived embryos contain maternal mitochondrial DNA (mtDNA) in oocytes. The mutation of mtDNA could cause cellular dysfunction, cancer, and diseases [38–40], limiting the potential uses of pluripotent cells obtained from this imperfect oocytes. the success of the replacement of mitochondrial genome in mature non-human primate It was recently reported [41]. This approach was performed by transferring spindle chromosomal complex from one egg to an enucleated, mitochondrial replete egg. The reconstructed oocytes with the mitochondrial replacement were capable of supporting normal fertilization, embryo development, and healthy offspring. Even this technique is skillful; it could be soon applied to human oocytes in which mutations of mtDNA are found.

Reprogramming by nuclear transfer technique has not been extensively demonstrated in human system since the access to a source of human oocytes is not only a rare opportunity, but also an ethical concern of the moment [42]. Apart from low availability, this procedure also depends on voluntary donation of these oocytes and the success rate of this technique is considerably low [43, 44]. More recently, somatic nuclei were transplanted into enucleated zygotes leading to the generation of cloned embryonic stem cells and mice. In this technique, mouse zygotes were temporarily arrested in mitosis using drug nocodazole. The resulting embryos developed into mice, thus supporting somatic nuclear reprogramming [45]. Application of this process toward human system may be applicable; however, recently, the translation of this procedure is restricted in human. Interspecies cloning, transplanting human somatic cell nucleus into enucleated animal oocytes, has shed light on surmounting the limitation of availability of human oocytes. In 2007, there was an introduction of controversial proposal in the UK for permitting the creation of interspecies embryos that was hoping to obtain a sufficient number of hybrid hESC lines for research [46]. But this had brought a massive argument toward the possibility of human clone generation, and in some countries this option had been completely banned. Moreover, due to the development of alternative approaches of nuclear reprogramming, human interspecies cloning is no longer in focus by scientists.

## 5. Heterokaryon: The Generation of Hybrid Pluripotent Cells

Another method to generate human pluripotent cells is cell fusion between somatic cells and hESCs [47] which is leading to the birth of “heterokaryon.” The term heterokaryon means a cell that contains multiple genetically different nuclei. In contrast to enucleated oocytes, ESC cytoplasm lacks the ability to reprogram somatic cells, but does the ESC nuclei [48]. A conceptually related approach to the reprogramming of somatic cell nuclei after cell fusion is the dominant genotype/phenotype of hESCs to nuclear reprogram of somatic cell after hybridization. The hybrid cells established by cell fusion show remarkably similar characteristics to hESCs. It was postulated that the cellular contents of hESCs comprised of reprogramming factors that could influence the epigenetic status of the nucleus of somatic cells back to pluripotent state [49, 50]. These reprogrammed cells expressed key pluripotent genes, Oct4, Sox2, and Nanog [47], and also could generate all three embryonic germ layers both *in vitro* and *in vivo *[51]. As this process involves the fusion of both somatic and embryonic stem cells, the reprogrammed cells consist of chromosomal components from both cells. It was found that neural stem cells were cocultured with ESCs suggesting that the acquisition of pluripotency by the adult neural stem cells may be mediated by spontaneous cell fusion with the pluripotent cells. These cells were found to fuse and retain both adult markers and pluripotential [52, 53]. The pluripotency of such a fusion hybrid cell may not be derived entirely from the somatic genome, as the somatic genome may be controlled by key regulatory genes of pluripotent cell genomes. 

However, it is challenging that since hybrid cells originate from the fusion of somatic cells and hESCs, the reprogrammed cells contain chromosomal materials from both cell types as well as the cells exhibit chromosomal tetraploid. Up to date, there has been no reported of the success to remove genetic contents of hESCs from the hybrids cells. It was hoping that if the hESC genomes were successfully removed from the hESC-somatic cell hybrids, it could be possible to obtain immune-matched pluripotent cells from patient's own cells. The use of hESC cytoplasm nucleated from the metaphase stage is another possible option [54]. However, with all these possibilities, there is still one note of caution that has to be seriously taken into account. Although the hybrids could contribute to all three embryonic germ layers, further investigations regarding their epigenetic anomaly are necessary. It needs to be clarified whether differentiated progeny from hybrid cells are functional and do not present any genetic/epigenetic aberrations which could subsequently result in cell transformation. Finally, the factors responsible for the regulation of somatic nuclei in fusion hybrids must be characterized before they find their usefulness in therapeutic approaches.

## 6. Induced Pluripotency by Transcription Factors: The Modulation of the Core Gene Regulatory Network

The breakthrough in iPSC research was due to the initial cloning experiment of Dolly the sheep [55]. Then, some studies revealed that reprogramming factors in ovular cytoplasm and ESCs are able to induce pluripotency in somatic cells [56]. In 2007, it was discovered that overexpression of 4 key transcription factors, Oct4, c-Myc, Sox2, and Klf4, of hESCs could reprogram human fibroblasts to hES-like cells, called human induced-pluripotent stem cells (hiPSCs) [16]. The success of iPSCs was confirmed by latter studies from multiple groups, including a combination of different factors, Nanog and Lin28 [57]. iPSCs have passed the most stringent examinations for gene expression profile, pluripotency, self-renewal and germ layer differentiation both *in vitro *and *in vivo* [16, 58], confirming their remarkable similarity to hESCs. The development of hiPSC technology and their characteristics have opened for the new hope for regenerative medicine. However, the recent report discovered that hiPSCs still retained epigenetic memory of the origin cell [59] and this could cause differentiation propensity of iPSCs toward the lineage of starting cells. This issue seems to limit further application of hiPSCs, especially cell-based therapy. 

In the past few years, major focuses for the derivation of human pluripotent stem cells are centered at iPSCs. Although the process of reprogramming by overexpression reprogramming factors sounds simple, there are currently two main problems with the iPSC technology: (1) the efficiency of hiPSC is substantially low which is less than 0.1% of fibroblasts become hiPSCs [16, 58, 60, 61] and (2) the use of virus as a vector can result in the random integration of viral DNA into the host-cell genome. Compared to other strategies, the use of viral vector, retrovirus, and lentivirus is considered as a high-efficient tool for gene transfer; thus, viral transfection is becoming the most popular choice for the expression of pluripotency-inducing transgenes [16]. However, the transfer of reprogramming genes by viral vectors appears to be a disadvantage of iPSCs in human clinical setting, such as transplantation, because the integration of viral DNA into the host-cell genome could alter the normality of host-cell genome and lead to cell transformation [62]. There are many studies to find alternatives to using viral vectors for reprogramming somatic cells. These techniques include the use of plasmid transfection and the piggyBac transposition system [63]. An alternative non-viral vector transfection method is using a single multiprotein expression vector comprised of coding sequences of c-Myc, Klf4, Oct4, and Sox2 linked with 2A peptides to reprogram both mouse and human fibroblasts [61]. Once reprogramming was achieved, the transgene could be removed using transient Cre expression. The piggyBac system is a transposon to deliver genes in mammalian cells. The piggyBac system is able to deliver large genetic elements without significant reduction in efficiency. This system was used to reprogram human fibroblasts to iPSCs [60]. Studies also explored the application of modified mRNA and proteins, encoded by those reprogramming genes [64, 65] and showed the success of reprogramming process by using these molecules. Nevertheless, compared to viral vectors, the proficiency of these alternatives significantly reduced the number of reprogrammed cells and the production of these reprogramming molecules requires specific laboratories. Despite many challenges, there are potentially multiple advantages of hiPSCs over hESCs, as the former can be generated from individual, maintaining one's genetic constitution and identity. In addition, iPSCs represent a source of differentiated cell types genetically identical to the person of origin that may be useful for screening drugs for individual forms of pathology and for being a source of transplantable tissues.

## 7. Future Challenges

It is a long and amusing history of nuclear reprogramming in which increasingly sophisticated technologies have became accessible. A comparison of pluripotent cells derived from these three different approaches exhibits certain common properties, including gene expression profile and differentiation capability. In addition, the elongation of telomere and reactivation of human telomerase reverse transcriptase are commonly found in these reprogrammed cells. However, for all nuclear reprogramming strategies, some somatic cells are more readily reprogrammed than others. Thus, these features can be differentially exploited to investigate the principle mechanisms underlying pluripotency. Each of the three approaches is distinct and has its own pros and cons. For instance, SCNT is characterized by rapid reprogramming process [66] and is ideally appropriated to elucidating the fundamental principles of early embryonic development and reproductive biology, as well as yielding a sufficient number of hESCs for therapeutic purposes. In contrary, cell fusion is technically simple. When cell fusion is used to form mixed-species heterokaryons, which do not proliferate, pluripotency genes is activated quickly and with high efficiency [67]. This approach is therefore particularly well suited to demonstrating molecular mechanisms that control the onset of nuclear reprogramming, but it does not yield clinical grade cells. On the other hand, forced expression of pluripotent transcription factors could provide such cells. Due to the ease of iPSC technology, it is now worldwide studied in laboratories all over the world. In addition, iPSCs offer a tool to study human diseases [68] and drug discovery [69]. In the near future, it is much possible that the novel approach of nuclear reprogramming will emerge which could present as the safer strategy and with higher efficiency than those currently available.

## Abbreviations

hESC: Human embryonic stem cell
ICM: Inner cell mass
MEF: Mouse embryonic fibroblast
SCNT: Somatic cell nuclear transfer.
